# INTRODUCTION TO SOMMA: CONCEPTUAL CONSTRUCT AND PARTICIPANT CHARACTERISTICS

**DOI:** 10.1093/geroni/igac059.831

**Published:** 2022-12-20

**Authors:** Steven Cummings, Peggy Cawthon, Theresa Mau, Stephen Kritchevsky, Russell T Hepple, Paul Coen, Bret Goodpaster, Anne Newman

**Affiliations:** San Francisco Coordinating Center, San Francisco, California, United States; California Pacific Medical Center Research Institute, San Francisco, California, United States; California Pacific Medical Center, San Francisco, California, United States; Wake Forest School of Medicine, Winston-Salem, North Carolina, United States; University of Florida, Gainesville, Florida, United States; AdventHealth, Orlando, Florida, United States; AdventHealth, Orlando, Florida, United States; University of Pittsburgh, Pittsburgh, Pennsylvania, United States

## Abstract

SOMMA aims to characterize key muscle characteristics and their longitudinal association with declines in mobility. To assess mitochondrial energetics in muscle, respiration assays were performed on biopsies of the vastus lateralis and the capacity to generate ATP (ATPmax) was determined by 31P MR spectroscopy. Other properties examined in biopsies include denervation, fiber type and size, capillary density and oxidative damage. We archive ~150mg for ancillary science. In a subset, we obtained biopsies of subcutaneous adipose for measurements including cell senescence and for archiving. To assess body composition, SOMMA used whole body MR for quadriceps volume and D3 Creatine dilution for total skeletal muscle mass. We measured fitness by cardiopulmonary exercise testing. Participants also had many other assessments of physical and cognitive performance including chair stands, balance, gait speed, leg power, stair climbing, Trails B, MOCA, and patterns of physical activity by actigraphy.

